# Supplementary material for: Chromothripsis during telomere crisis is independent of NHEJ, and consistent with a replicative origin
Source: Genome Res. 2019 May;29(5):737–49. doi: 10.1101/gr.240705.118 (PMC6499312; doi:10.1101/gr.240705.118)
Supplement: Supplemental Material [file supp_gr.240705.118_Supplemental_file_1.zip › contigs/annotated_contigs/DB79/contig.2.DB79_length_655_mean_cov_7.32824427481.docx]

**DB79_length_655_mean_cov_7.32824427481**

```
TATTTAATATGAATACTTCACATCAACCTGAATTGATAATGAGATATCTACCATAGCATAACACATTGACCTTGTAAAATACATCAACT
 >chr2:142642189-142642613 - E=2e-243
GCCTCCTCTGAGCCAATCAATCCATCCAAATACATTTACAGGTAAATTTTGTTTTCTGATGAATCATTTTTCAGCTGATTTCCTATTCA

CCGGGTTAGTGTGACCTCAAAGATAGTTTGCTTTATCTTATAAACATTAAGATACTTTATGCAGCATGAGGCTGCAAACAGAATGTAGG

GGCAATAATCTCTTCTGTGAGGGCAATTTGAGGAAGACTATCCACATCGGCCCATTCATAGTGACTTTTAGCTATGACATTCCAGACAA

ATGTTAAATATGTTCAGACATTGTCAACTGACAACATTTTAACATGAGTATGTTTAAAAGATTAT|TCT|TTTTAGAAGTGAAGTTTTG
                                                                  >chr2:142766915-14276
CAGAAACAACAGAATTGAGTGAGAGGATCCCATACAACACGTGCAAAAATCTGAAGGCCTAGCACCTTATAGAGAAATGCTGTATGTCA
7149 - E=5e-129
GTGTGTCAGGAGGGCAGGATGCATGTGAAGTATCAATGCAAGACGGTGCTTCAGAGATAGGCAAGAGTCAACTACTTTATCAGTAAACT

TCAACATGAGGAGAGGGTGAAAAAGGACATCTCG
```
